# Engineering the ferroelectric polarization to optimize the GIDL and negative output conductance in negative capacitance FET

**DOI:** 10.1038/s41598-025-18041-7

**Published:** 2025-10-03

**Authors:** Vijay Sai Thota, Sandeep Moparthi, Kalyanbrata Ghosh

**Affiliations:** School of Electronics Engineering, Vellore Institute of Technology, Vellore, Tamil Nadu 632014 India

**Keywords:** Negative differential resistance, GIDL, Negative capacitance, Ferroelectric, Industry, Innovation, Infrastructure, Electronics, photonics and device physics, Electrical and electronic engineering

## Abstract

This paper presents the optimization of the gate induced drain leakage (GIDL) and negative output conductance (NOC) effect in ferroelectric negative capacitance (NC) FET by engineering the polarization of ferroelectric. The improvement in NOC and GIDL is presented with reworked polarization and hence the channel potential at the drain side through gate workfunction modulation. An NC SOI device structure is considered in this work, as this study is intended to investigate the proposed optimization technique. With the metal length ratios of 1:1 and 1:2, the proposed structure offered an enhanced ferroelectric polarization via surface potential amplification at the drain end of the channel. The onset of NOC is delayed by 0.21 V with an improved GIDL current of 3.18 nA and a minimum subthreshold swing of 33.45 mV/decade for the metal length ratio of 1:1.

## Introduction

Ferroelectric negative capacitance (NC) FETs are promoted as a feasible option for achieving a steep subthreshold slope, enabling faster switching and low-power design^1–4^. The ferroelectric offers NC during its polarization transition. When placed in the gate stack of MOSFET, the NC can enhance the MOSFET’s overall gate capacitance/control. This, in turn, results in gate voltage amplification in MOSFET^5–8^. Unlike conventional FET, NC FET offers negative drain-induced barrier lowering (N*DIBL)* besides a steep subthreshold slope. However, the *NDIBL* of NC FET manifests the negative output capacitance (*NOC*)^9,10^, which can be observed in the output characteristics of the device. The *NOC* has resulted from the deterioration of ferroelectric polarization at the drain end of the device at higher drain voltages^10^. The poor gate voltage control at higher drain voltage hampers ferroelectric polarization reversal and its negative capacitance. As a result, the channel potential of NC FET dampens at the drain side when the drain voltage is higher, and hence, the drain current falls, which can be seen in the output characteristics. Though the steep switching of NC FET is desired in digital circuits, the *NOC* is not desirable for analog applications, as it de-amplifies the intrinsic gain. There are methods proposed in the literature to optimize the *NOC* in NC FET. An asymmetric source and drain parasitic capacitance approach is proposed to tune the *NOC*, which adjusts the capacitance matching between the baseline FET and ferroelectric^11^. Apart from this, the material parameters of ferroelectric, namely remnant polarization and coercive field, are tuned to optimize the *NOC* of NC FET^12^. In the literature, it is also shown that the *NOC* increases with a decrease in the ratio of remnant polarization (*P*_*r*_) to the coercive field (*E*_*c*_) in a ferroelectric material^12^. However, the material parameters *P*_*r*_ and *E*_*c*_ of ferroelectric (primarily doped-HfO_2_ based) are greatly influenced by the fabrication process, such as annealing temperature and doping concentration.

Our group proposed reducing the *NOC* in NC Silicon nanotube FET by increasing its core gate radius^13^. This approach minimises the *NOC* by tuning the capacitance matching^13^. Though the proposed method is more suitable for gate-all-around nanowires and nanotubes, it leads to an increase in the effective radius of the channel. A gate underlap at the drain side is proposed in the literature to minimize drain dominance on the gate and, hence, the better *NOC*^14^. However, this approach increase the device length and limit the gate control over the channel. A local Gaussian heavy doping (pocket) at the top surface of the channel near the drain is proposed to improve the channel potential at the drain end^15^. In this approach, it is crucial to control the doping profile of the channel, and it also affects the carrier mobility^15^. In the literature, it is proposed to introduce a charge trapping layer between the ferroelectric and dielectric layers of the gate stack^16^. The induced trap charges near the drain side enhance the surface potential and hence the better *NOC*. However, introducing an extra trap charge layer in the gate stack influences the capacitance matching of the device^16^.

In addition to NOC, the NC FET exhibits a poorer GIDL than its conventional counterpart due to the steep energy band profile. The literature proposes that lightly doped source and drain regions improve the GIDL of the NC FET by altering the steep profile of the energy band diagram^17^. However, this approach deteriorates the device’s drive current due to the limited carrier availability in the source^17^. A gate-drain overlap is proposed in the literature^18^ to control the GIDL of NC tunnel FET with enhanced gate control. However, it requires a precise control of overlap/underlap of gate at nano scale.

The aforementioned literature mainly focused on tuning capacitance matching in NC FET to optimize the *NOC*. However, these proposals in the literature lead to more delay owing to higher parasitic capacitances. As per the studied literature, there are very limited works focused on the GIDL of NC FET which is a major contributor to the standby leakage. Moreover, it is essential to combinedly analyse and optimize the impact of GIDL and NOC on NC FET to make it more adaptable to both analog and digital applications. With these observations, this paper proposes a surface potential modulation approach through dual metal (DM) workfunction to improve the *NOC* and *GIDL* with channel potential amplification at the drain end. An SOI device structure is considered for investigation in the proposed optimization technique. The following sections of this paper explain the device structure and simulation methodology, followed by the discussion and conclusion.

## Device structure and simulation methodology

**Fig. 1:**
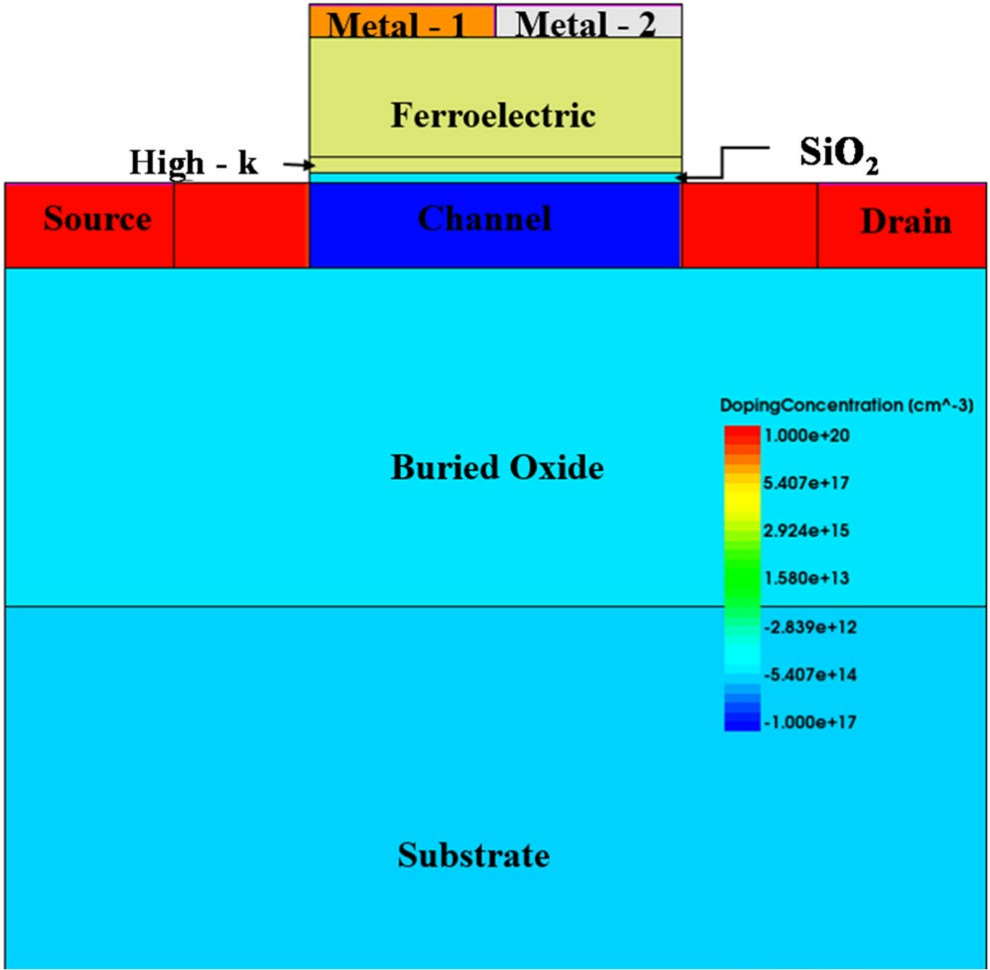
Device structure of DM NC SOI FET.

**Fig. 2:**
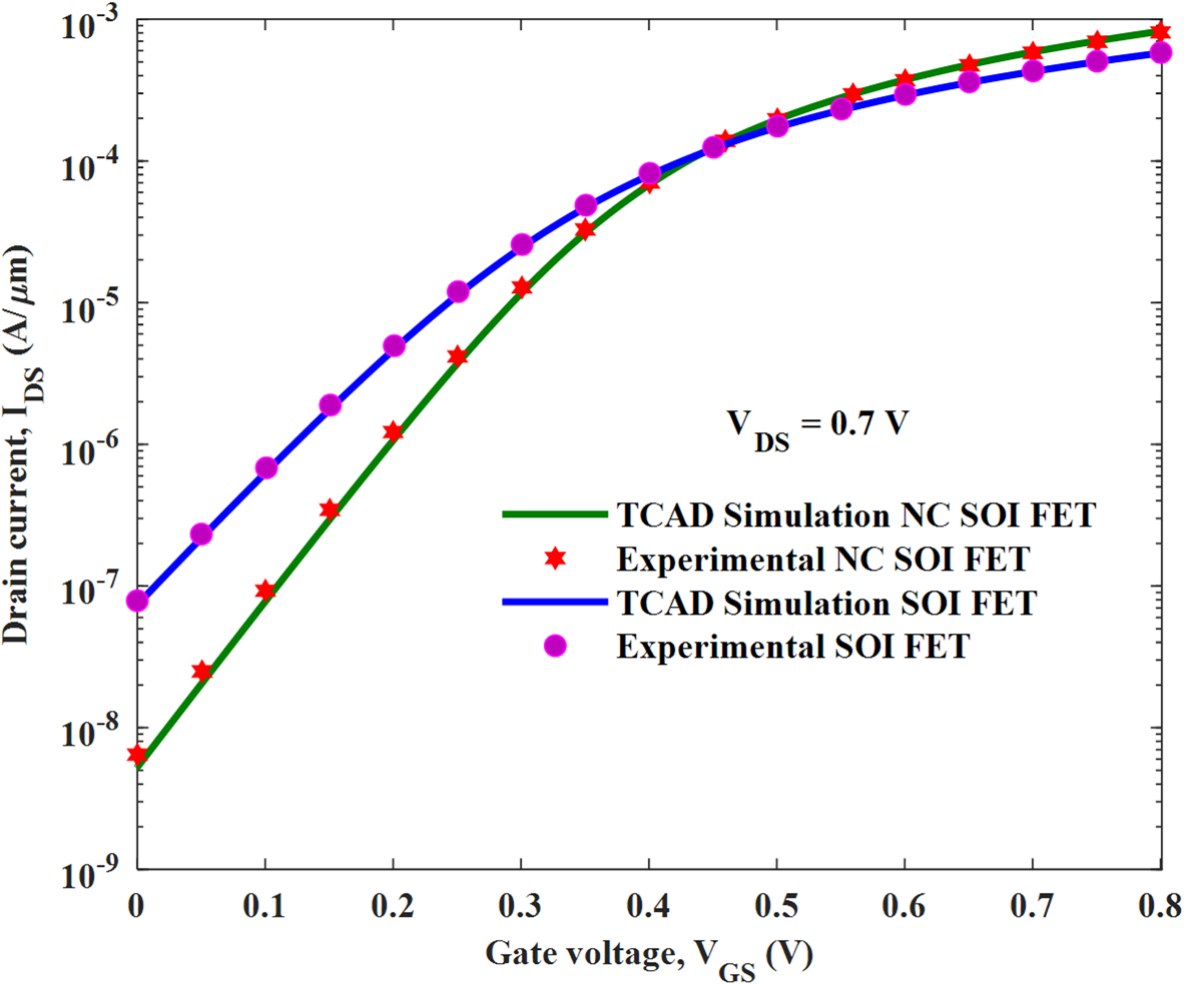
Validation of TCAD simulation models for NC SOI FET.

A 2D NC SOI FET structure is considered in this work, as this study is intended to investigate the proposed optimization technique. The considered device structures are simulated using Sentaurus TCAD from Synopsys. The device structure and its parameters are shown in Fig. 1; Table 1, respectively. To account for the quantum confinement effects at a channel thickness of 5 nm, QuantumPotential model of Senataurus TCAD is used. The doping dependent (Philips unified), high field saturation, Enormal (Lombardi, inversion and accumulation layer), OldSlotboom, Bandgap Narrowing model, and Schenk bandgap models are used in the simulation. The ferroelectric polarization model is invoked in the ferroelectric layer. Figure 2 shows the calibration of the simulation methodology as per Fig. 1 (d) of^19^. This is based on the experimental data of ^20^ for SOI FET and with the ferroelectric P_r_ and E_c_ values extracted from the experimental P-E curve of ^21^ for NC SOI FET. The values of the ferroelectric material parameters are calculated as α = −1.1e11 cm/F, β = 2.5e21 cm^5^/FC^2^ from the extracted P_r_ and E_c_ values of 14.83µC/cm^2^ and 1.25MV/ cm, respectively. The coupling coefficient of ferroelectric polarization gradient (g) and viscosity (ρ) are considered as 1e-4 cm^3^/F and 0.18 Ω.cm, respectively.

**Table 1:** Device parameters.

Parameter	Values
Channel length (*L*)	22 nm
Channel doping (*N*_*A*_)	10^17^ cm^−3^
Channel thickness (*t*_*si*_)	5 nm
Gate oxide thickness (*t*_*SiO2*_)	0.9 nm
High-k dielectric thickness (*t*_*high-k*_)	1.2 nm
Ferroelectric thickness (*t*_*fe*_)	7 nm
Gate-metal workfunction	Metal-1 (*Φ*_*M1*_): 4.65 eV Metal-2 (*Φ*_*M2*_): 4.5 eV

## Result analysis

The result analysis is carried out as follows. Section A presents a comparative study of the Single Metal (SM) NC SOI FET and the conventional SM SOI FET. Section B presents the NOC and GIDL optimization of the NC SOI FET with surface potential modulation.

### Comparative study of SM NC and conventional SOI fets

**Fig. 3:**
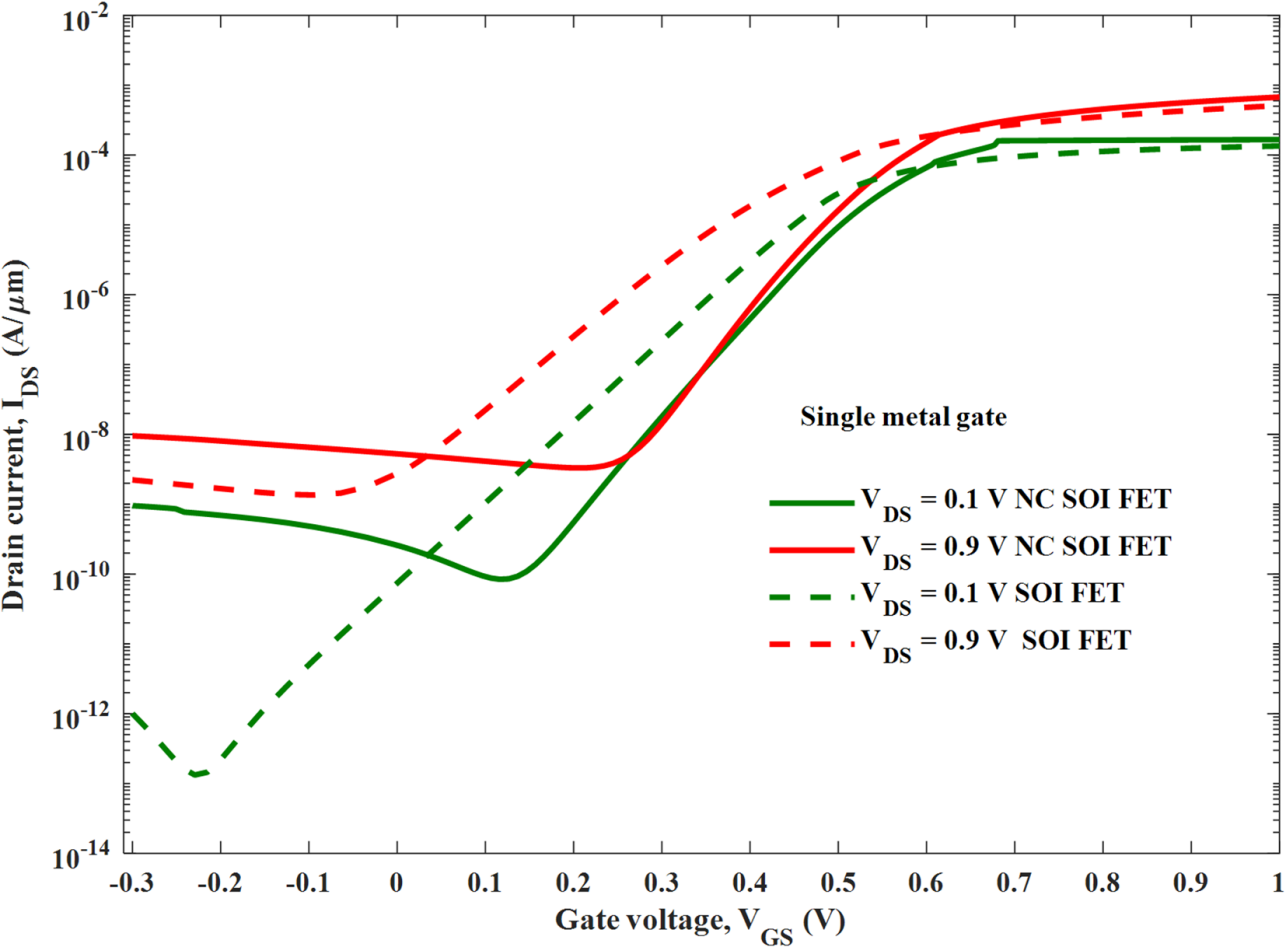
Transfer characteristic of SM SOI FET and SM NC SOI FET.

The transfer characteristics of the SM SOIFET are plotted with and without NC, as shown in Fig. 3. Electrical metrics of NC SOI FET and SOI FET are tabulated in Table 2. The analysis shows improved SS, I_ON_, and threshold (V_th_) voltage roll-up in NC SOI FET, which can be attributed to the negative capacitance induced by the ferroelectric. It is seen that, despite steep subthreshold slope, the SM NC SOI FET exhibited measurable leakage current due to GIDL (I_GIDL_) compared to conventional SOI FET at V_GS_=-0.3 V and V_GS_=0 V for the V_DS_ of 0.1 V and 0.9 V, as shown in the Table 3.

**Table 2:** Electrical metrics comparison of SOI FET and NC SOI FET.

	SS_average_ (mV/decade) at V_DS_ of		V_th_ (V) at V_DS_ of		I_ON_ (mA) at V_DS_ of	
	0.1 V	0.9 V	0.1 V	0.9 V	0.1 V	0.9 V
SOI FET	86	96	0.433	0.413	0.13	0.50
NC SOI FET	70	79.13	0.636	0.548	0.166	0.67

**Table 3:** GIDL comparison of SOI FET and NC SOI FET.

	SM NC SOI FET (I_GIDL_)		SM SOI FET (I_GIDL_)	
	V_DS_ = 0.1 V	V_DS_ = 0.9 V	V_DS_ = 0.1 V	V_DS_ = 0.9 V
V_GS_ = − 0.3 V	9.5e − 10 A	9.5e − 9 A	9.73e − 13 A	2.22e − 9 A
V_GS_ = 0 V	2.57e − 10 A	5.26e − 9 A	7.39e − 11 A	2.79e − 9 A

**Fig. 4:**
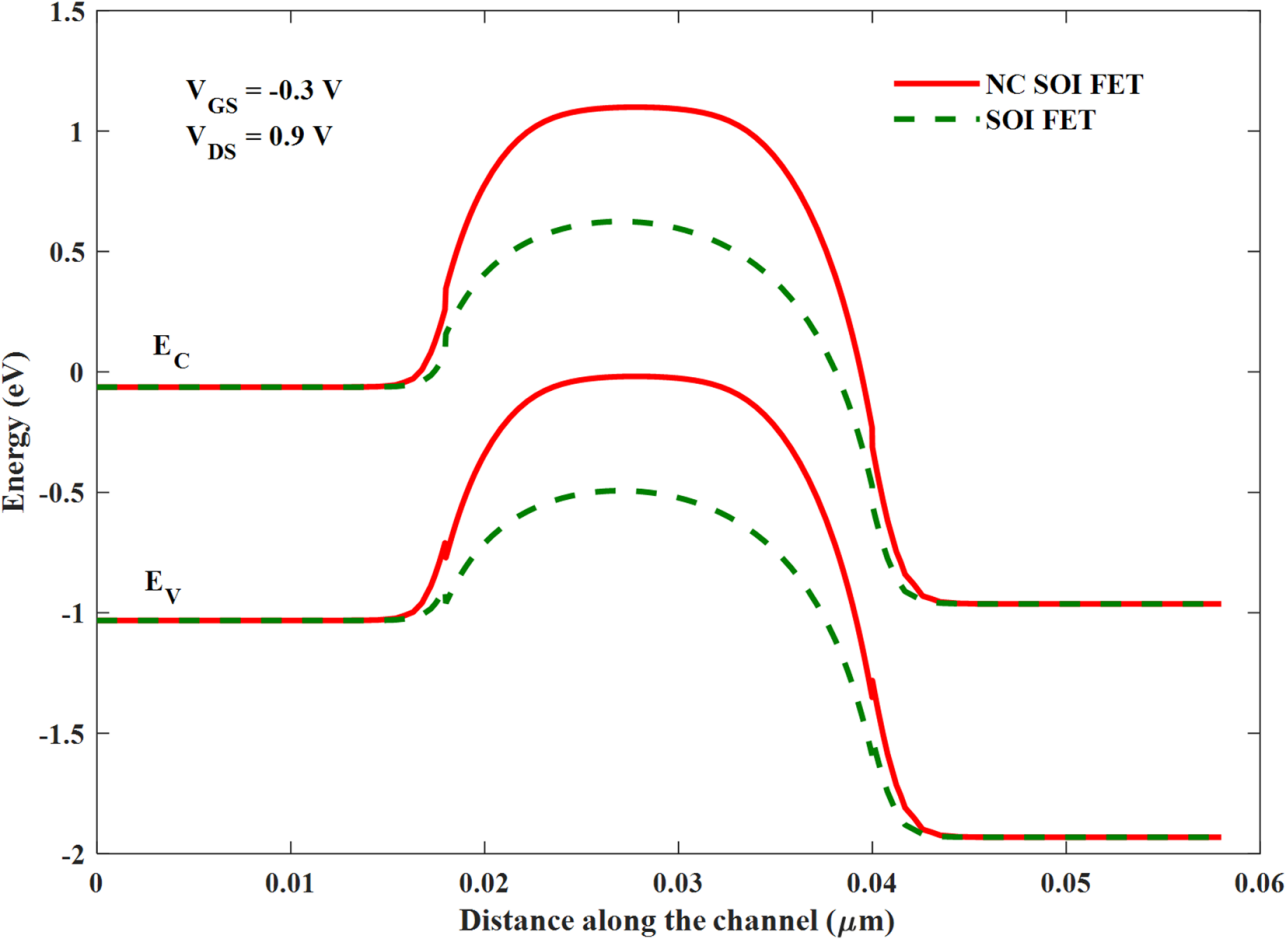
Energy band profile of SM SOI FET and SM NC SOI FET.

This is due to the gate-to-drain leakage caused by the steeper energy band profile of NC FET (especially at the drain end), as shown in Fig. 4. The shift in the threshold voltage of NC SOI FET observed in Fig. 3 is analyzed along with surface potential modulation, as depicted in Fig. 7.

**Fig. 5:**
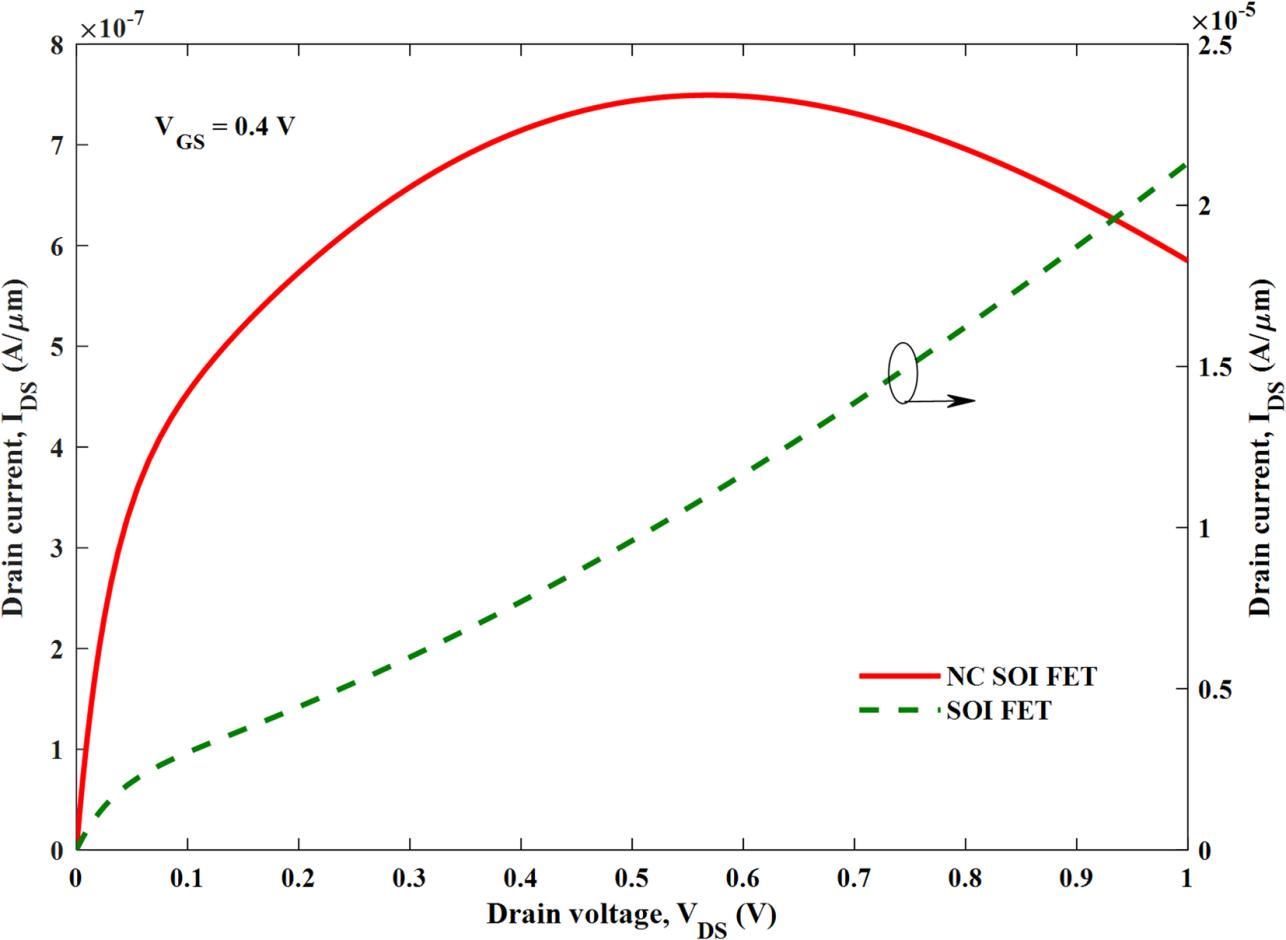
Output characteristics of the SM SOI FET and NC SOI FET (NOC in NC SOI FET despite significant short channel effect in conventional SOI FET).

**Fig. 6:**
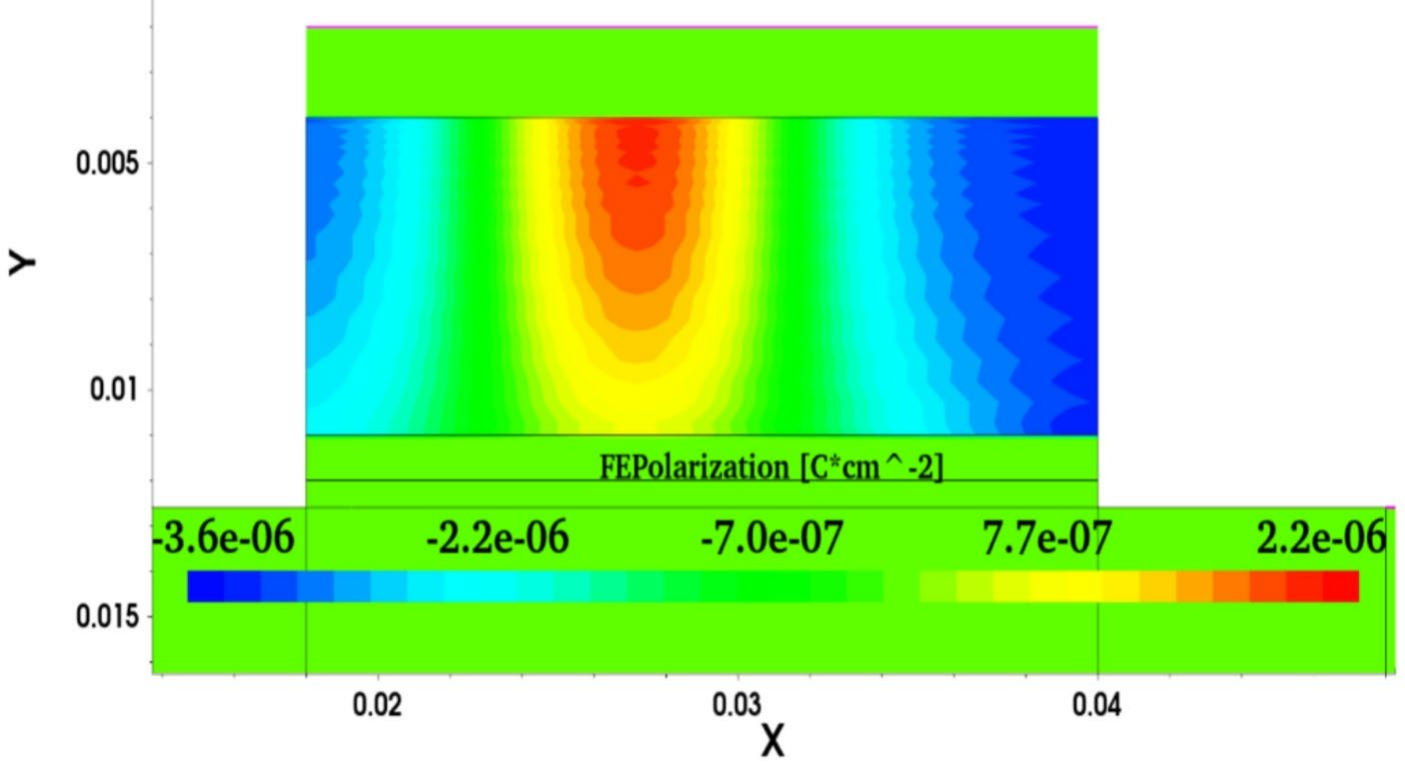
Ferroelectric polarization of the SM NC SOI FET at V_GS_ = 0.4 V and V_DS_ = 0.9 V along the channel.

**Fig. 7:**
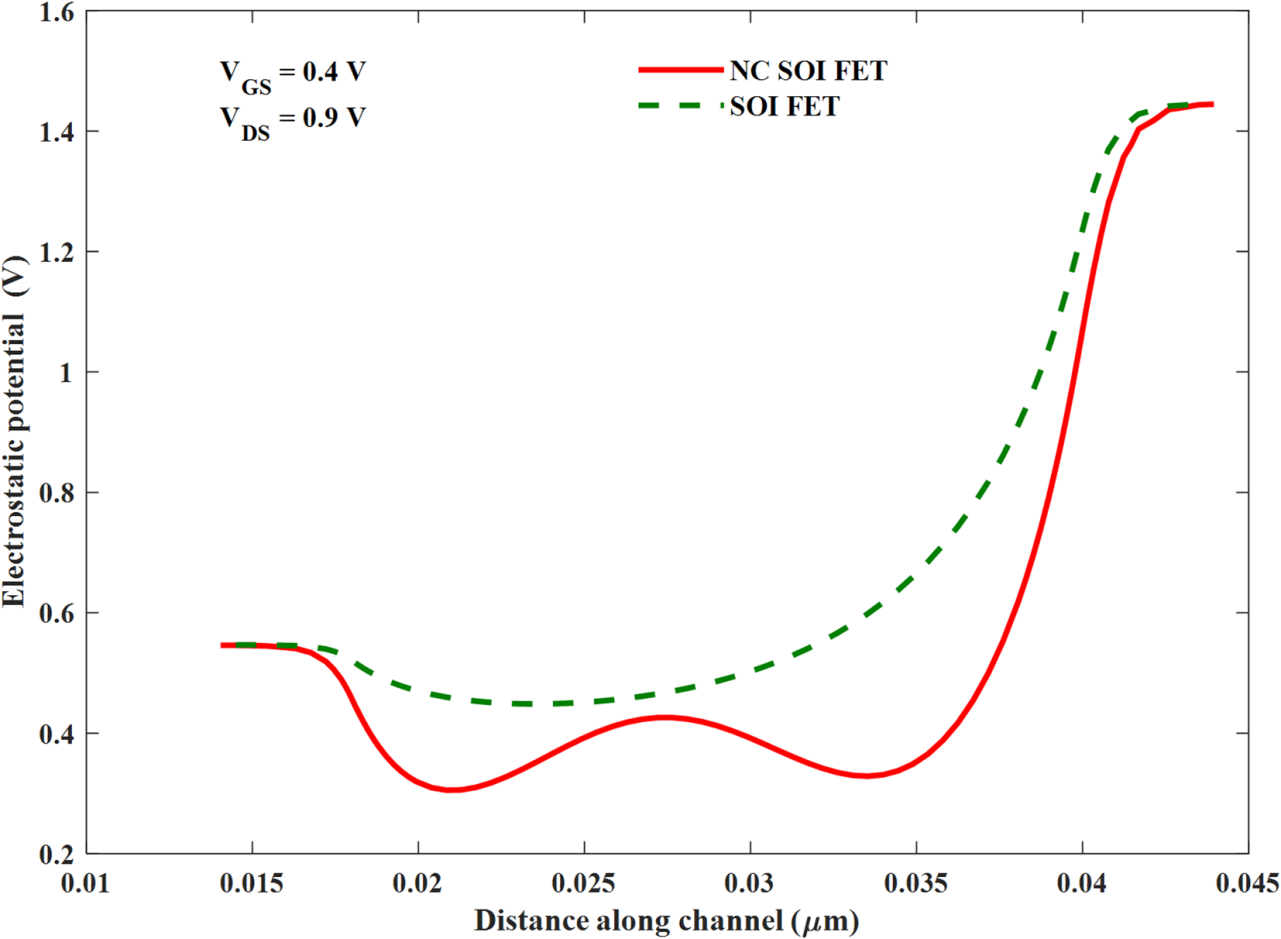
Surface potential of the SM SOI FET and NC SOI FET.

Moreover, the SM NC SOI FET depicted the negative output conductance (NOC), which is unlike a conventional device, as seen from the output characteristics in Fig. 5. It is worth noting that the NC SOI FET exhibited the NOC despite a very high saturation current observed in its conventional counterpart owing to the short-channel effect. The NOC in the NC SOI FET is due to dampened ferroelectric polarization at the drain side, as shown in Fig. 6, owing to poor gate control at higher drain voltages. This dampened polarization of NC SOI FET resulted in the depression of the surface potential of SM NC SOI FET compared to conventional SOI FET, as shown in Fig. 7. This depressed surface potential, in turn led to the increment in the threshold voltage of NC SOI FET as shown in Fig. 3. The aforementioned rise in threshold voltage of the NC SOI FET can also be observed in Fig. 4 in terms of the height of the energy band profile. As the aforementioned results show, NC SOI FET suffers from GIDL and NOC despite improved DIBL compared to conventional SOI FET.

### Optimization of NOC and GIDL in NC SOI FET with surface potential modulation

**Fig. 8:**
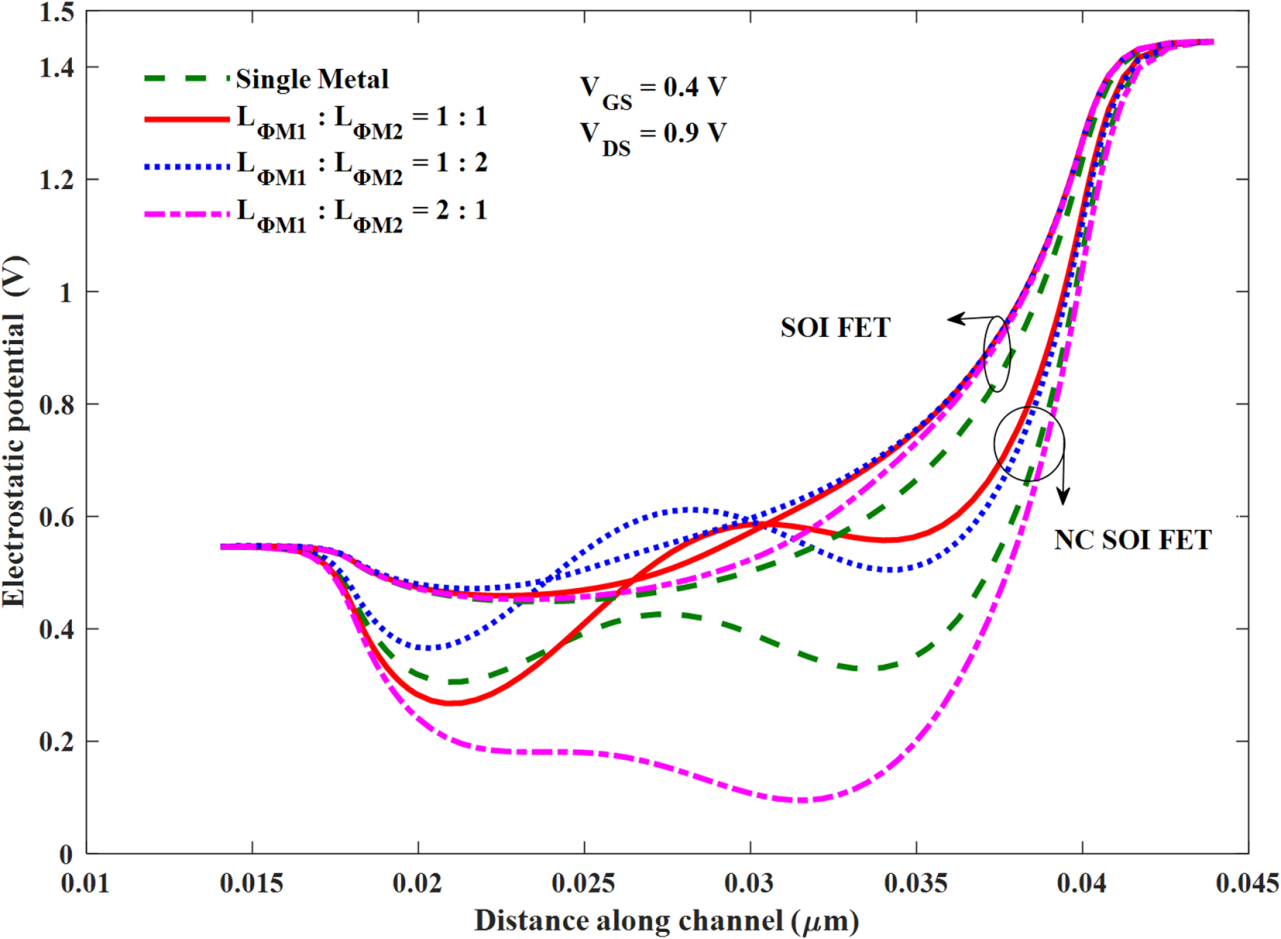
Surface potential of the SM and DM gate SOI FET and NC SOI FET.

The following section of the results presents the optimization of the GIDL and NOC of NC FET through the modulation of the height of the energy band and surface potential along the channel by gate metal workfunction engineering. The surface potential profiles (along the channel) of the NC SOI FET and conventional SOI FET are compared in Fig. 8 for SM and DM gates. The gate workfunction at the drain side of the channel is lowered to amplify the surface potential and thereby increase the ferroelectric polarization in NC SOI FET through better gate control at higher drain voltages. For the DM FETs, the workfunction of the gate is considered as *ɸ*_M1_=4.65 eV and *ɸ*_M2_=4.5 eV at the source side and drain side, respectively. The ratio of the lengths of the DM gate (*L*_*ɸM1*_:*L*_*ɸM2*_) is taken as 2:1, 1:1, and 1:2.

**Fig. 9:**
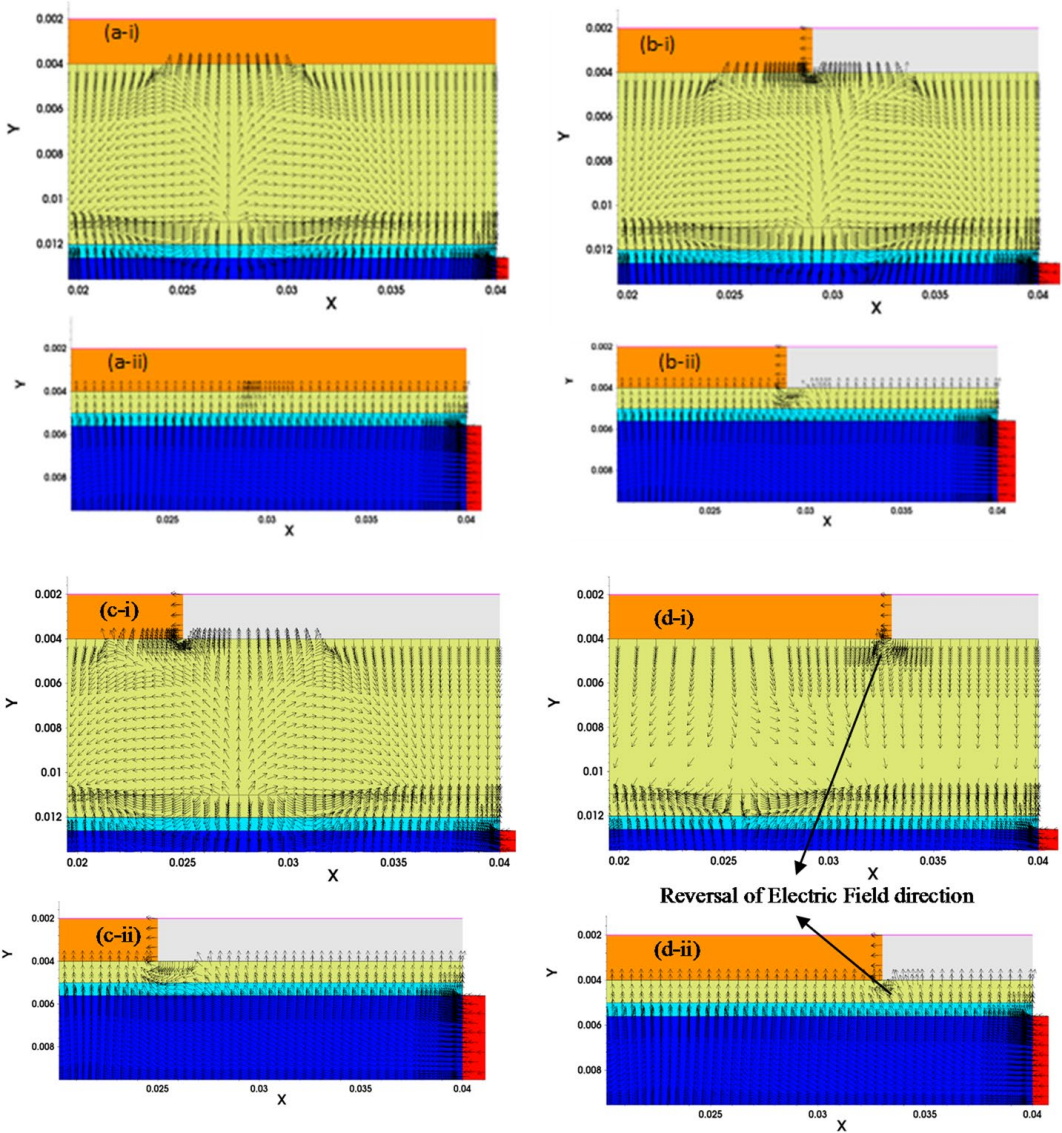
Electric field vector representation of: (i) NC SOI FET and (ii) SOI FET for (**a**) SM, (**b**) *L*_*ɸM1*_:*L*_*ɸM2*_ = 1:1, (**c**) *L*_*ɸM1*_:*L*_*ɸM2*_ = 1:2, and (**d**) *L*_*ɸM1*_:*L*_*ɸM2*_ = 2:1 at V_GS_ = 0.4 V and V_DS_ = 0.9 V.

**Fig. 10:**
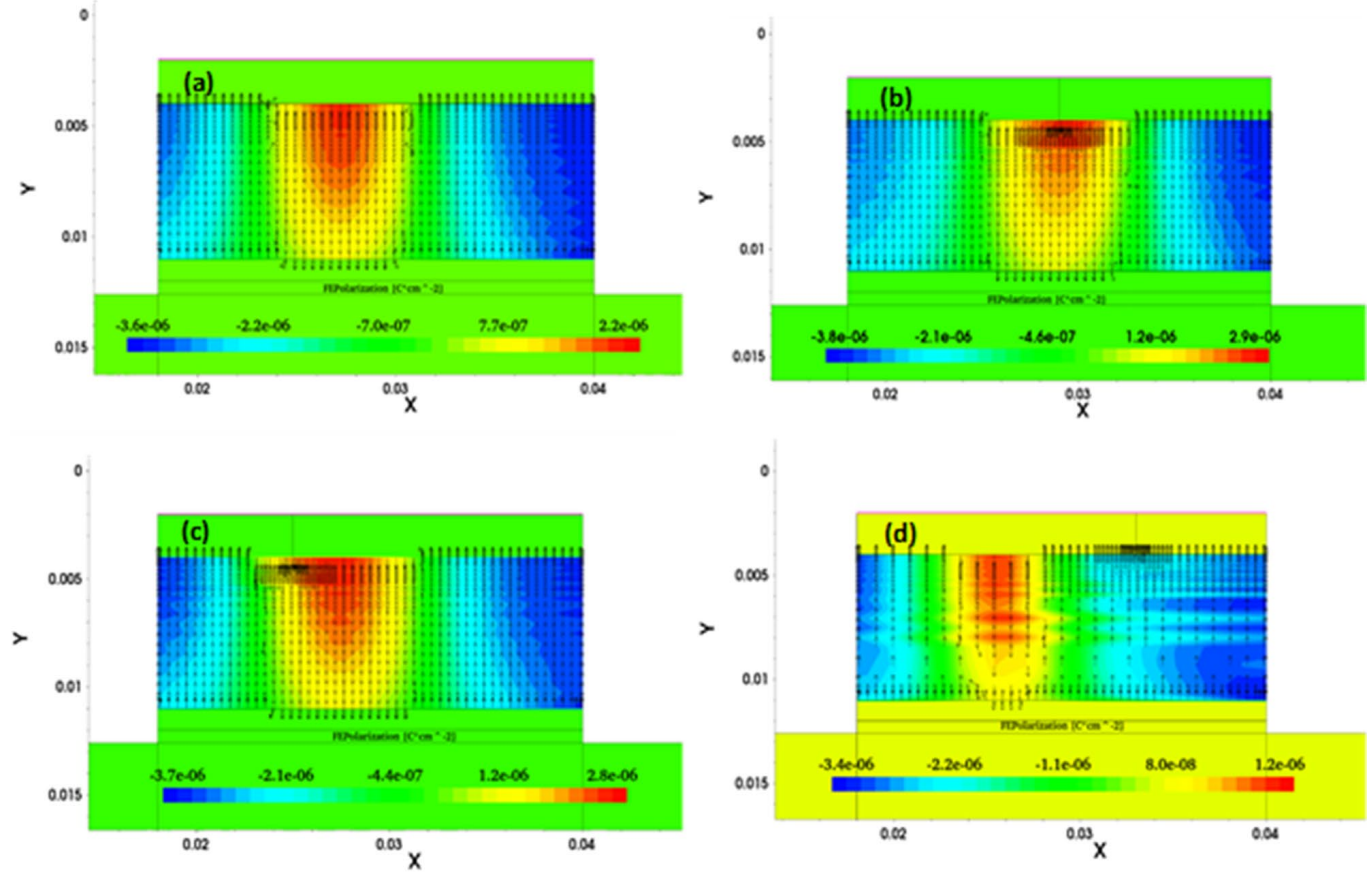
Ferroelectric polarization contour with its vector representation of the NC SOI FET along the channel (**a**) SM and DM for cases of *L*_*ɸM1*_:*L*_*ɸM2*_ of (**b**) 1:1, (**c**) 1:2, (**d**) 2:1 at V_GS_ = 0.4 V and V_DS_ = 0.9 V.

As seen from Fig. 8, the surface potential of the conventional SOI FET is increased proportionally along the length of the gate with workfunction ɸ_M2_ (*L*_*ɸM2*_). This, in turn, makes the device conduct early due to the channel inversion at the lower gate voltage (threshold voltage). On the other hand, it is observed from the surface potential profiles of the NC SOI FET that the trend is similar to that of the conventional SOI FET, except for the 2:1 case. The reason for the anomaly in the 2:1 case of NC SOI FET can be understood with the help of the electric field and ferroelectric polarization profiles shown in Figs. 9 and 10, respectively. From Fig. 9 (d-i, ii), it is observed that the electric field direction of the NC SOI FET with *L*_*ɸM1*_:*L*_*ɸM2*_ of 2:1 is in the opposite direction to the electric field of the SOI FET. This contrasts with the electric field directions observed in the remaining cases of *L*_*ɸM1*_:*L*_*ɸM2*_ shown in Fig. 9.

This abnormality in the electric field direction of NC SOI FET for the *L*_*ɸM1*_:*L*_*ɸM2*_ of 2:1 is attributed to the extended polarization dampening of ferroelectric at the drain end, as seen from Fig. 10 (d). In NC SOI FET with *L*_*ɸM1*_:*L*_*ɸM2*_ of 2:1, the interface potential of gate metals aids the drain voltage since the interface of *ɸ*_*M1*_ and *ɸ*_*M2*_ is located near the drain. Therefore, the gate control at the drain end of the NC SOI FET with *L*_*ɸM1*_:*L*_*ɸM2*_ of 2:1 further weakens, which results in poor ferroelectric polarization and hence surface potential.

**Fig. 11:**
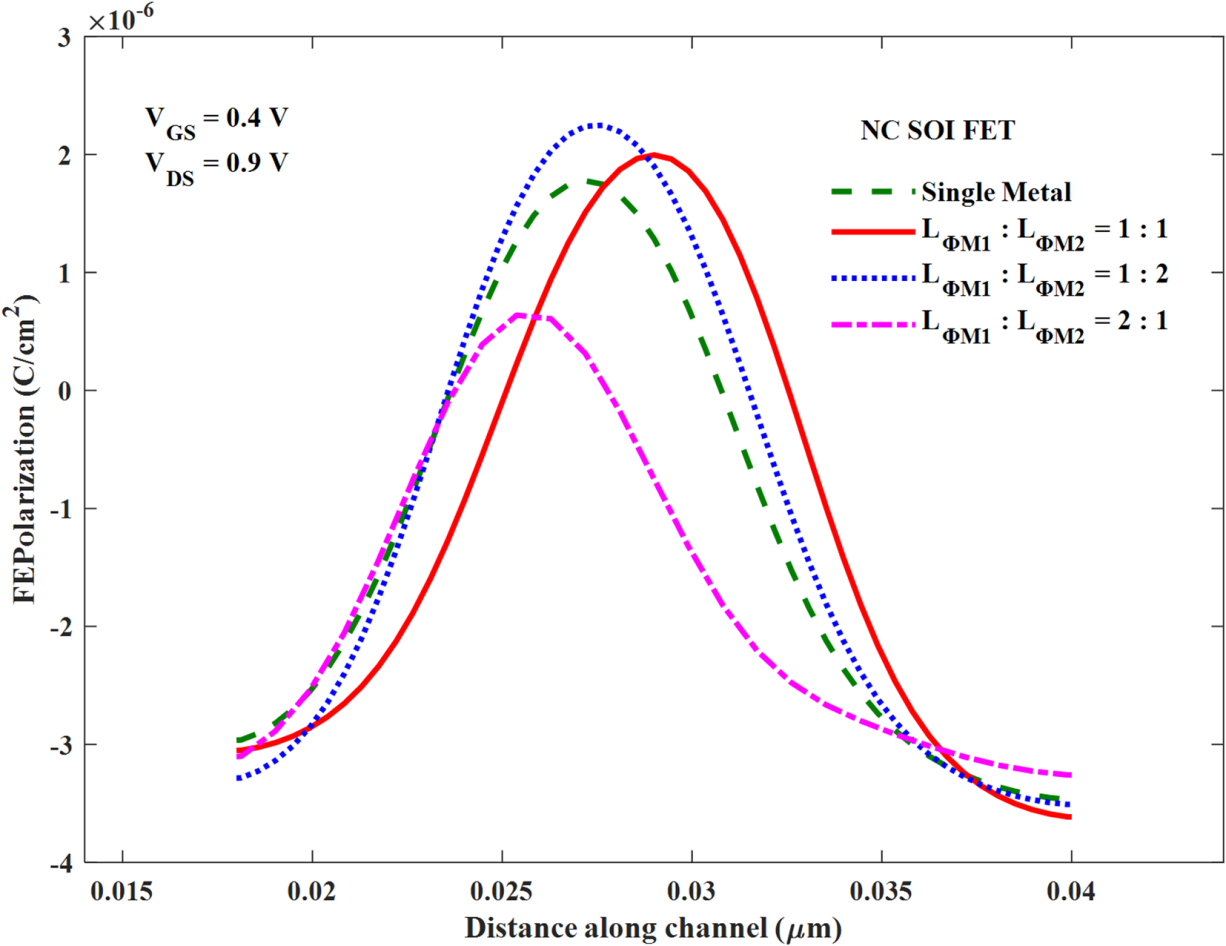
Comparison of ferroelectric polarization along the channel of NC SOI FET.

Whereas in the other cases of *L*_*ɸM1*_:*L*_*ɸM2*_, polarization dampening (at the drain end) of DM NC SOI FET is controlled compared to SM, as seen in Fig. 10 (a-c). The ferroelectric polarization along the channel is compared in Fig. 11 for all cases of *L*_*ɸM1*_:*L*_*ɸM2*_ with SM SOI FET, which depicts the above observations of polarization dampening in the case of *L*_*ɸM1*_:*L*_*ɸM2*_ of 2:1 and improved polarization in 1:1 and 1:2 cases.

**Fig. 12:**
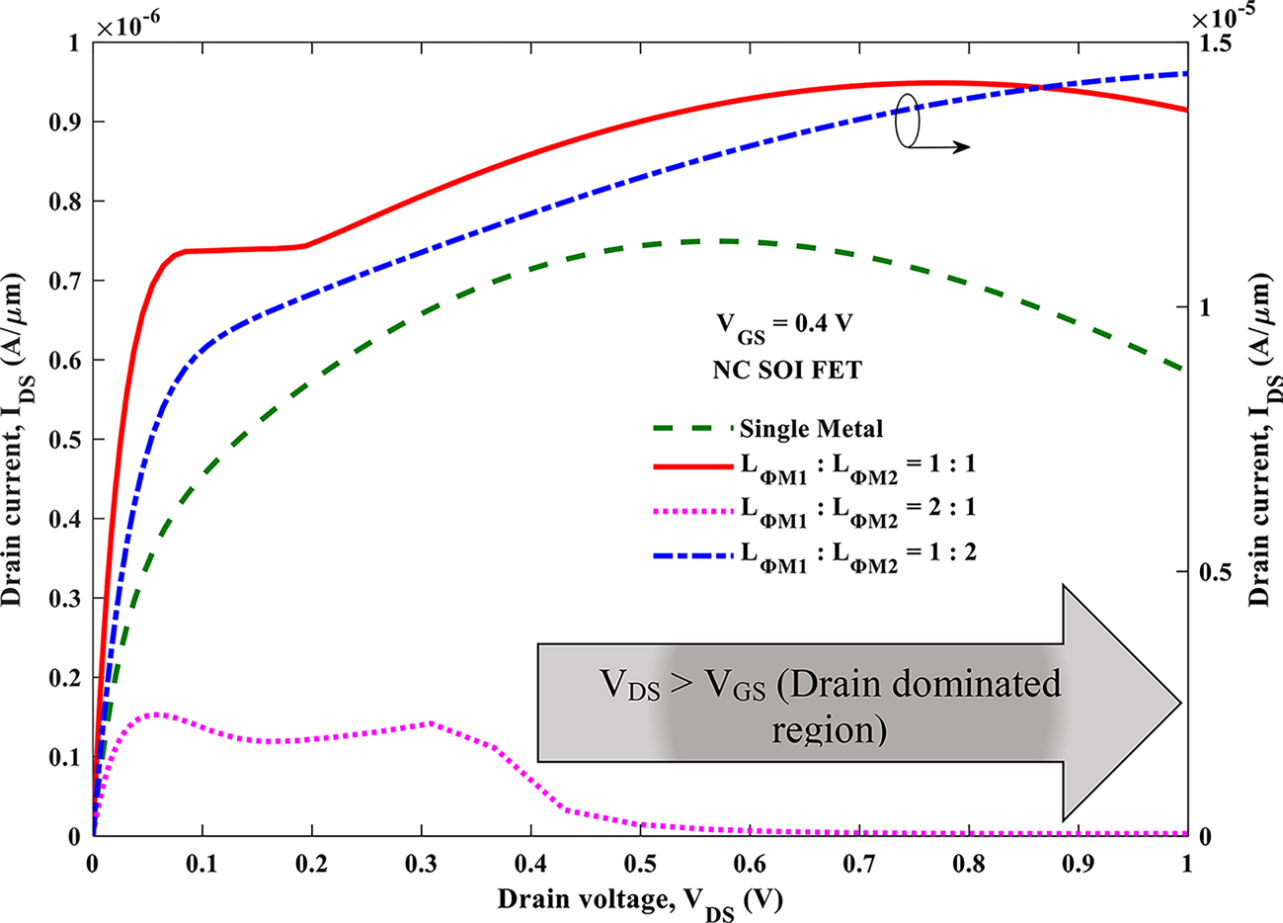
Output characteristics of SM and DM NC SOI FET.

**Fig. 13:**
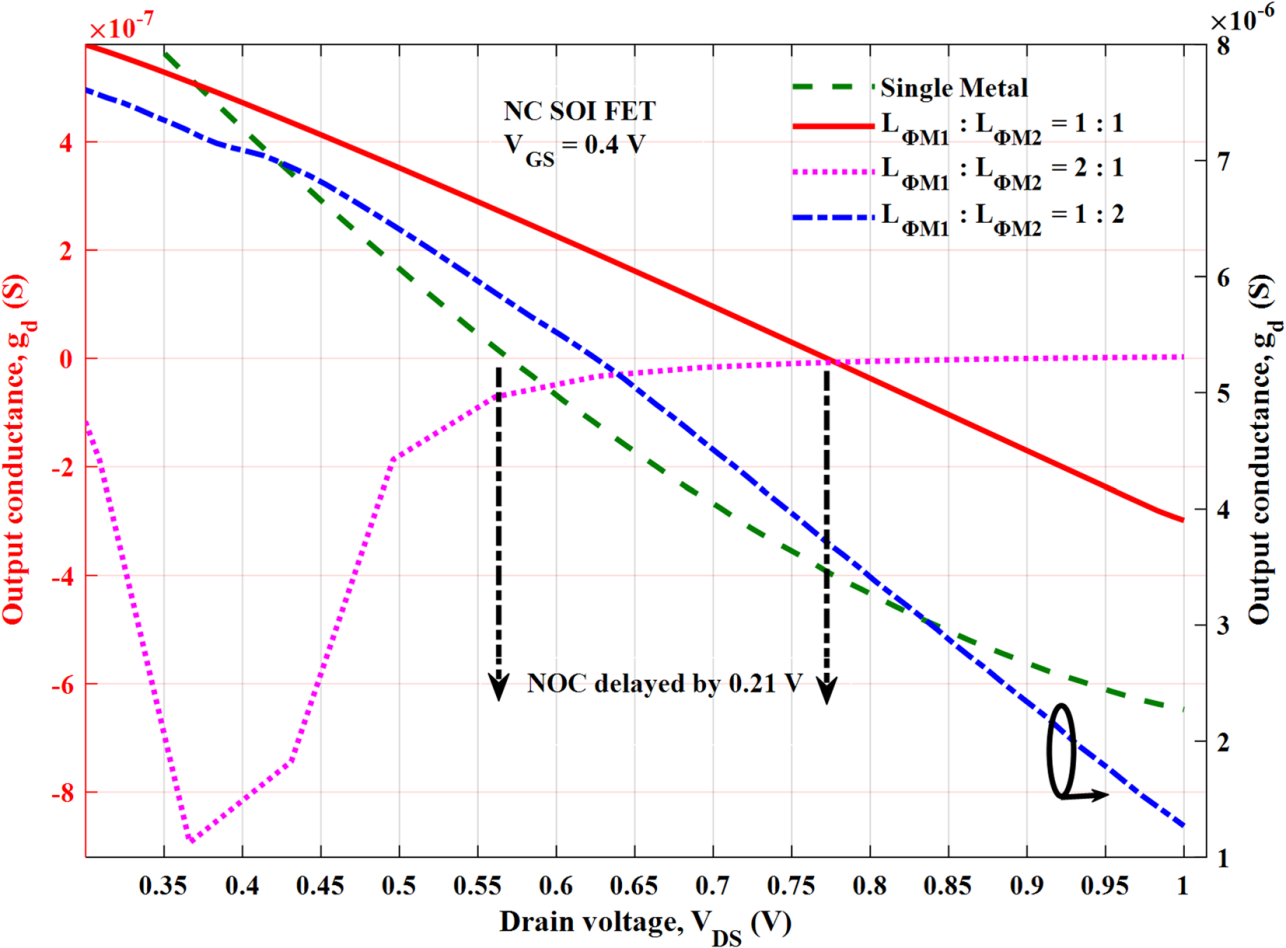
Output conductance of SM and DM NC SOI FET.

The output characteristics of the NC SOI FET are depicted in Fig. 12 at a gate voltage of 0.4 V for different *L*_*ɸM1*_:*L*_*ɸM2*_. It is clear that the SM NC SOI FET suffers from the NOC despite short-channel effects, as discussed in Fig. 5. The improvement in the negative slope of output characteristics i.e. NOC is seen with *L*_*ɸM1*_:*L*_*ɸM2*_ of 1:1 and 1:2. The same trend is reflected in the output conductance as shown in Fig. 13, for the respective combinations of *L*_*ɸM1*_:*L*_*ɸM2*_. In case of *L*_*ɸM1*_:*L*_*ɸM2*_ of 1:1, the occurrence of NOC is observed at a V_DS_ of 0.77 V, which is delayed by 0.21 V compared to V_DS_ of 0.56 V for SM NC SOI FET. Besides, in the case of *L*_*ɸM1*_:*L*_*ɸM2*_ of 1:2, NOC is mitigated as seen in Figs. 12 and 13.

The aforementioned improvement in NOC can be attributed to improved ferroelectric polarization as seen from Fig. 10 (b, c). However, for *L*_*ɸM1*_:*L*_*ɸM2*_ of 2:1, the NOC is further deteriorated than SM NC SOI FET due to poor ferroelectric polarization as observed in Fig. 10 (d). From Figs. 12 and 13, it is required to note that the NOC for *L*_*ɸM1*_:*L*_*ɸM2*_ of 2:1 is more prominent at the drain voltages greater than the gate voltage of 0.4 V due to weaker gate control.

**Fig. 14:**
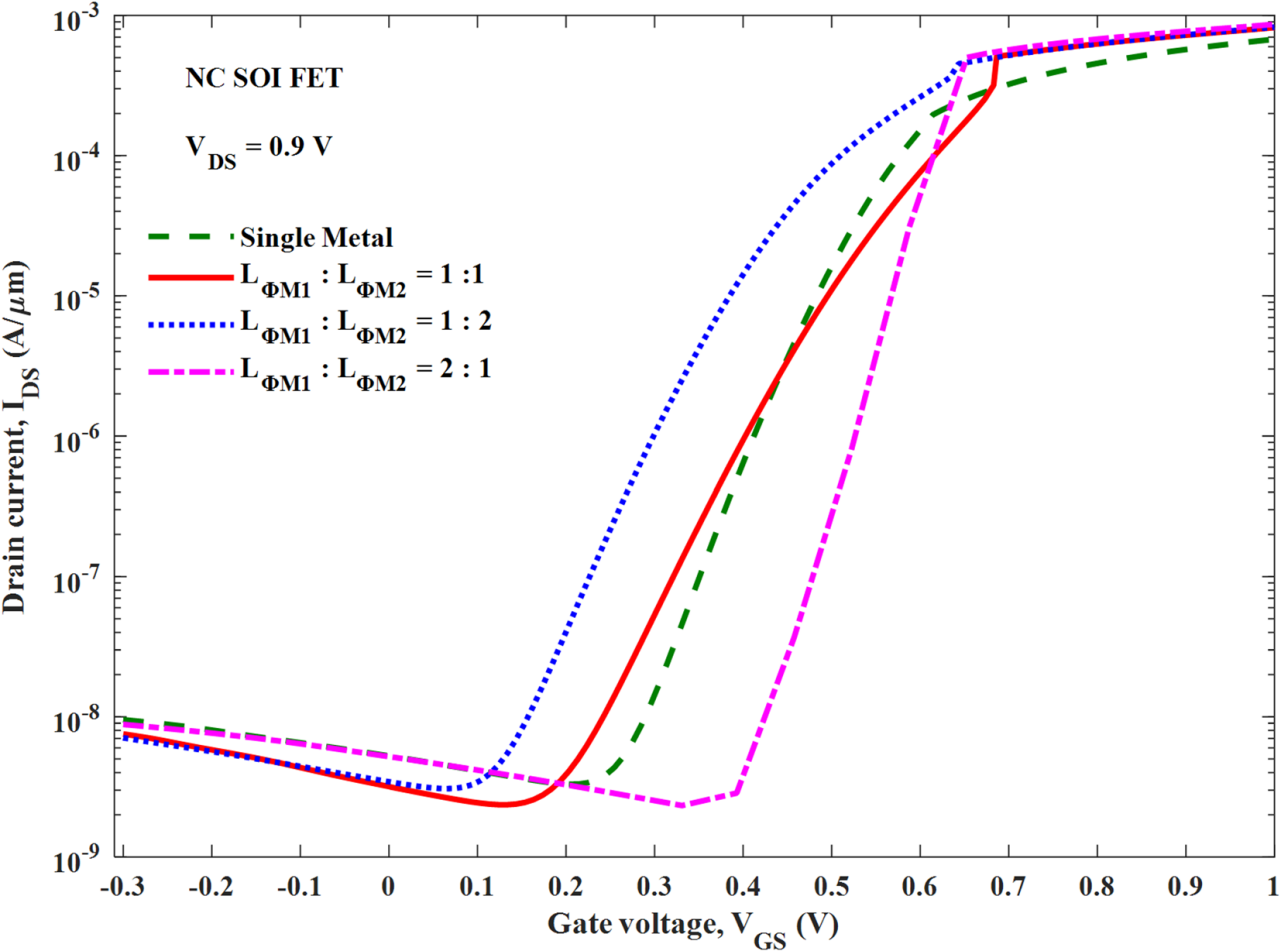
Transfer characteristics of SM and DM NC SOI FET.

**Fig. 15:**
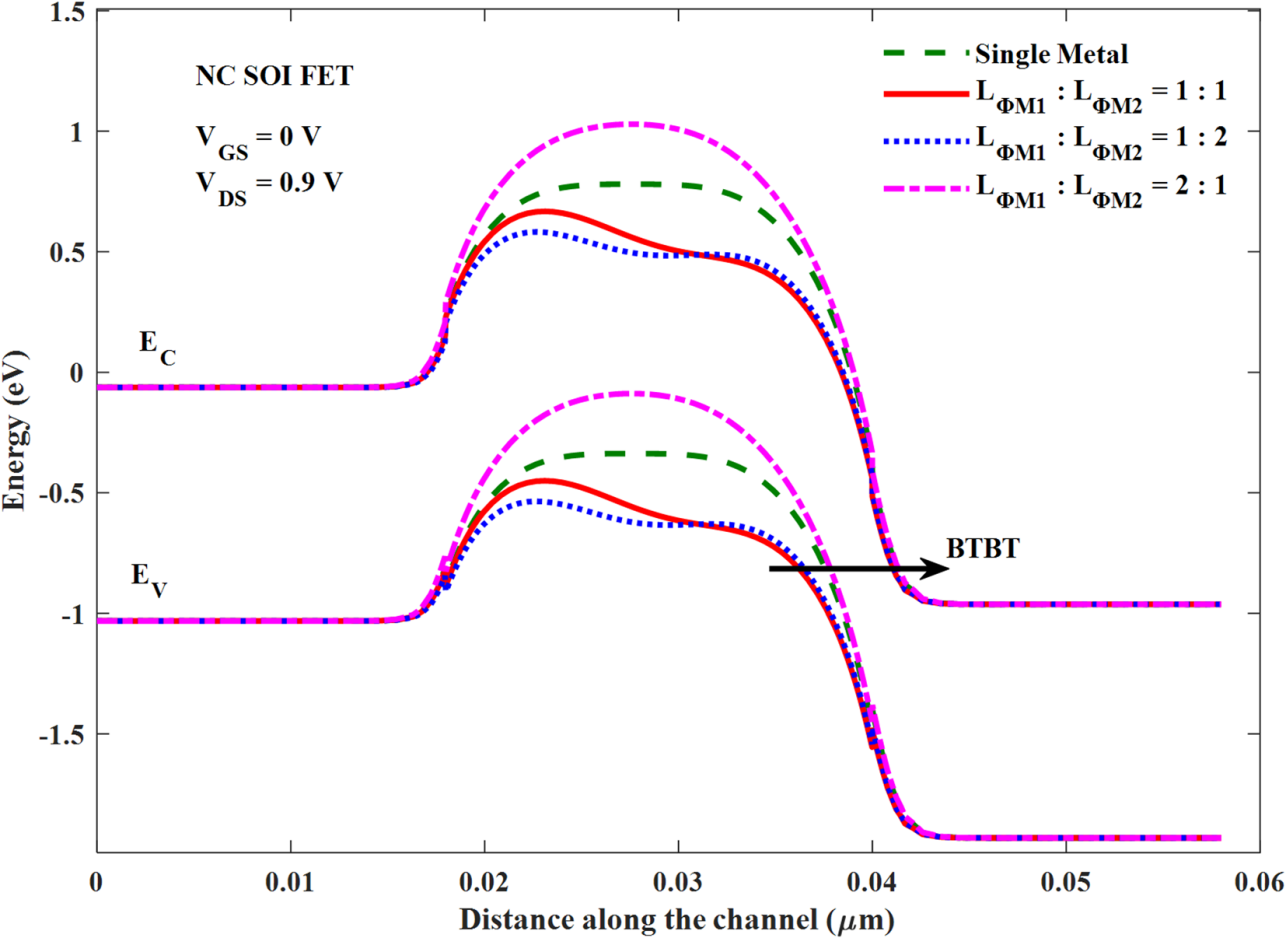
Energy band profile of SM and DM NC SOI FET.

The transfer characteristics are plotted in Fig. 14 for the considered combinations of *L*_*ɸM1*_:*L*_*ɸM2*_. From Fig. 14; Table 4, it is observed that the *L*_*ɸM1*_:*L*_*ɸM2*_ of 1:2 offered poor subthreshold swing (SS) of 125.87 mV/decade compared to the remaining cases, though there is no NOC seen in Figs. 12 and 13. Whereas the *L*_*ɸM1*_:*L*_*ɸM2*_ of 1:1 offered the best average and minimum SS of 75.49 mV/decade and 33.45 mV/decade respectively, besides it’s improved *NOC* depicted in Fig. 12. On the other hand, *L*_*ɸM1*_:*L*_*ɸM2*_ of 2:1 offered a moderate improvement in SS compared to SM NC SOI FET. However, its NOC is measurably deteriorated as discussed from Figs. 12 and 13. The energy band profiles of the considered combinations of *L*_*ɸM1*_:*L*_*ɸM2*_ are plotted in Fig. 15 at a V_GS_ of 0 V. As observed from Figs. 14 and 15; Table 4, *L*_*ɸM1*_:*L*_*ɸM2*_ of 1:1 has the best I_GIDL_ of 3.18 nA due to a lesser extent of band to band tunnelling (BTBT) resulted from its comparatively wide band gap at the drain end. Furthermore, this work is compared with the literature based on performance metrics such as I_ON_, I_ON_/I_OFF_, NOC, and GIDL, as shown in Table 5. It demonstrates the mitigation/optimization of both NOC and GIDL, whereas other studies have focused only on optimizing NOC. The I_ON_ and I_ON_/I_OFF_ values of this work are consistent with the literature.

**Table 4:** Comparison of minimum SS, average SS, I_ON_, V_th,_ I_GIDL_ for SM and DM NC SOI FET.

L_ɸM1_:L_ɸM2_	SS_min_. (mV/decade) at V_DS_ = 0.9 V	SS_average_. (mV/decade) at V_DS_ = 0.9 V	V_th_ (V) at V_DS_ = 0.9 V	I_ON_ (mA) at V_DS_ = 0.9 V	I_GIDL_ (nA) at V_DS_ = 0.9 V V_GS_ = − 0.3 V	I_GIDL_ (nA) at V_DS_ = 0.9 V V_GS_ = 0 V
SM	58.79	79.13	0.548	0.674	9.51	5.26
1:1	33.45	75.49	0.672	0.818	7.52	3.18
1:2	67.50	125.87	0.574	0.824	7.06	3.44
2:1	44.45	73.25	0.65	0.868	8.812	5.199

**Table 5:** Comparison of the proposed NC SOI FET with the literature.

Device type	Methodology	L (nm)	T_FE_ (nm)	I_ON_ (mA/µm)	I_ON_/I_OFF_	NOC optimized/mitigated	GILD effect
NC SOI FET^12^	P_r_ = 6 µC/cm^2^ & E_c_ = 3 MV/cm	20	4	~ 0.8	~ 10^6^	Yes	Not analyzed
NC SOI FET^22^	FE layer at BOX &gate oxide	20	3	1.18	~ 10^5^	Yes	Not analyzed
NC SOI FET^22^	FE layer at gate oxide	20	3	1.05	~ 10^7^	No	Not analyzed
NC SOI FET^19^	With T_FE_ variation	20	7	~ 2.5	~ 10^6^	No	Not analyzed
NC SOI FET^19^	With T_FE_ variation	20	1.7	~ 1	~ 10^5^	Yes	Not analyzed
NC SOI FET (SM)	This work	22	7	0.674	~ 10^6^	No	Analyzed
NC SOI FET (*L*_*ɸM1*_:*L*_*ɸM2*_ of 1:1)	This work	22	7	0.818	~ 10^6^	Yes (optimized)	Analyzed & optimized
NC SOI FET (*L*_*ɸM1*_:*L*_*ɸM2*_ of 1:2)	This work	22	7	0.824	~ 10^6^	Yes (mitigated)	Analyzed & optimized

## Conclusion

The comparative performance analysis of NC SOI FET is carried out by tuning the ferroelectric polarization and surface potential of the channel through gate workfunction modulation. From the analysis, it is understood that the NC SOI FET with gate metal length ratio (*L*_*ɸM1*_:*L*_*ɸM2*_) of 1:1 offered an optimum trade-off between NOC, GIDL, and SS. Though the *L*_*ɸM1*_:*L*_*ɸM2*_ of 1:2 exhibited no NOC, its SS_min_ and SS_average_ are deteriorated to 67.5 mV/decade and 125.87 mV/decade, respectively. On the other hand, the NOC of NC SOIFET is greatly deteriorated at a gate metal ratio of 2:1 due to dampened ferroelectric polarization despite the improvement in SS_min_ and SS_average_ when compared to a SM metal NC SOI FET. The ON-OFF current ratio of NC SOI FET is increased in all cases of workfunction modulation. From the findings, it is concluded that the ferroelectric polarization can be tuned carefully through gate workfunction modulation due to its significant impact. However, this method has limited choice in short channel regime as it is difficult to control the gate workfunction variations.

## Data Availability

The datasets generated during and/or analysed during the current study are available from the corresponding author on reasonable request.
